# 

**Published:** 1887-07

**Authors:** 


COLLABORATORS:

CHICAGO:

PROFESSOR E. ANDREWS, M.D., LL.D
PROFESSOR J. BARTLETT, M.D.
PROFESSOR W. H. BYFORD, A.M., M.D.
PROFESSOR L. CURTIS, A.M., M.D.
PROFESSOR O. C. DEWOLF, A.M., M.D.
PROFESSOR E. C. DUDLEY, A.B., M.D.
PROFESSOR C. FENGER, M.D.
PROFESSOR E. WING, A.M., M. D.
PROFESSOR W. T. BELFIELD, M.D.
PROFESSOR J. N. HYDE, A.M., M.D.
PROFESSOR E. F. INGALS, A.M., M D
PROFESSOR W. W. JAGGARD, A.M., M.D.
PROFESSOR F. S. JOHNSON, A.M., M.D.
PROFESSOR H. A. JOHNSON, M.D., LL.D.
PROFESSOR C. W. PURDY, M.D.
PROFESSOR C. G. SMITH, A.M., M.D.
PROFESSOR F. BILLINGS, M.D.
PROFESSOR N. S. DAVIS, JR., A.M., M.D.

UNITED STATES ARMY:
SURGEON D. L. HUNTINGTON, M.D.

MARINE HOSPITAL SERVICE:
SURGEON S. T. ARMSTRONG, M.D.

MILWAUKEE, WIS.
PROFESSOR N. SENN, M.D.

WASHINGTON, D. C.:

S. O. RICHEY, M.D.

UNITED STATES NAVY:
MEDICAL DIRECTOR J. M. BROWNE, M.D.
MEDICAL DIRECTOR A. L. GIHON, A.M., M.D.

